# Aboveground carbon in Quebec forests: stock quantification at the provincial scale and assessment of temperature, precipitation and edaphic properties effects on the potential stand-level stocking

**DOI:** 10.7717/peerj.1767

**Published:** 2016-03-03

**Authors:** Louis Duchesne, Daniel Houle, Rock Ouimet, Marie-Claude Lambert, Travis Logan

**Affiliations:** 1 Direction de la recherche forestière, Ministère des Forêts, de la Faune et des Parcs, Québec, Canada; 2 Consortium sur la climatologie régionale et l’adaptation aux changements climatiques (Ouranos), Montréal, Canada

**Keywords:** Boreal forest, Biomass, Climate change, Carbon stock

## Abstract

Biological carbon sequestration by forest ecosystems plays an important role in the net balance of greenhouse gases, acting as a carbon sink for anthropogenic CO_2_ emissions. Nevertheless, relatively little is known about the abiotic environmental factors (including climate) that control carbon storage in temperate and boreal forests and consequently, about their potential response to climate changes. From a set of more than 94,000 forest inventory plots and a large set of spatial data on forest attributes interpreted from aerial photographs, we constructed a fine-resolution map (∼375 m) of the current carbon stock in aboveground live biomass in the 435,000 km^2^ of managed forests in Quebec, Canada. Our analysis resulted in an area-weighted average aboveground carbon stock for productive forestland of 37.6 Mg ha^−1^, which is lower than commonly reported values for similar environment. Models capable of predicting the influence of mean annual temperature, annual precipitation, and soil physical environment on maximum stand-level aboveground carbon stock (MSAC) were developed. These models were then used to project the future MSAC in response to climate change. Our results indicate that the MSAC was significantly related to both mean annual temperature and precipitation, or to the interaction of these variables, and suggest that Quebec’s managed forests MSAC may increase by 20% by 2041–2070 in response to climate change. Along with changes in climate, the natural disturbance regime and forest management practices will nevertheless largely drive future carbon stock at the landscape scale. Overall, our results allow accurate accounting of carbon stock in aboveground live tree biomass of Quebec’s forests, and provide a better understanding of possible feedbacks between climate change and carbon storage in temperate and boreal forests.

## Introduction

Over recent decades, increased human activities have affected many aspects of the Earth’s systems, through the increase of atmospheric CO_2_ concentration and associated warming of the planet surface (Smith et al., 2014). Along with human activity, forest ecosystems play an important role in the net balance of atmospheric CO_2_, acting as a carbon (C) sink for anthropogenic CO_2_ emissions (Trumper et al., 2009; Smith et al., 2014). Globally, the amount of C stored in soils and vegetation of forested ecosystems (approximately 860 Pg C, Pan et al., 2011) is nearly equivalent to the amount of C present in the atmosphere (approximately 851 Pg C; 399 ppm × 2.134 Pg C/ppm). On average, approximately 42% of the forest C stock is comprised in living vegetation biomass, 9% in dead wood, and the remaining (49%) in litter and soils (Pan et al., 2011). Since the end of the 20th century, established forests have captured more C than they have released, resulting in a net carbon sink of approximately 2.4 Pg C year^−1^ (2.3–2.5 Pg C year^−1^ depending on the period being considered), which is nearly equivalent to the amount of C captured by the oceans (2.3 Pg C year^−1^, Pan et al., 2011). This C sink corresponds to approximately 30% of the total anthropogenic C emissions due to fossil fuel combustion, cement production and land use change (8.4 Pg C year^−1^, Pan et al., 2011).

The amount of C found in live biomass of forest ecosystems (current aboveground carbon stock, CACS) depends on environmental conditions, life history attributes, morphological characteristics of tree species, disturbance regimes, and land-use history (Keith, Mackey & Lindenmayer, 2009). The CACS contained in live tree biomass is generally less than the carbon carrying capacity (CCC) in aboveground live biomass, which has been defined as “the mass of carbon able to be stored in a forest ecosystem under prevailing environmental conditions and natural disturbance regimes, but excluding anthropogenic disturbance” (Roxburgh et al., 2006; Keith et al., 2010).

While CACS of a given forest stand can be quantified relatively easily using forest inventory data and tree allometric equations, such is not the case for CCC, which is not known *a priori* and can only be estimated on the basis of given assumptions. In most studies, CCC of forest stands has been estimated from forest plots assumed to be representative of mature forests that were not significantly affected by recent large-scale disturbances and human activities (e.g., Keith, Mackey & Lindenmayer, 2009). The CCC of a forest stand can also be estimated with modeling approaches, but some limitations exist due to the scarcity of field data needed to calibrate the C model parameters (Roxburgh et al., 2006). However, when moving from the stand to the landscape scale, CCC estimates must take into account the natural disturbance regime which is adding considerable complexity to the estimation. In this paper, we use a different formulation that consists in the maximum stand-level aboveground carbon stock (MSAC) achievable under the prevailing environmental conditions without considering the influence of natural and human disturbances.

Some authors suggested that climate warming could increase the CCC (and consequently the MSAC) of forest ecosystems at the stand level (Anderson et al., 2006; Lewis et al., 2009). In addition to atmospheric CO_2_ concentration, climate is exerting a control on photosynthesis through the effects of temperature on carboxylation rates, stomatal conductance and nitrogen uptake (Woodward, Smith & Emanuel, 1995; Boisvenue & Running, 2006; Keith, Mackey & Lindenmayer, 2009). Air temperature also regulates many soil processes such as organic matter decomposition and mineral weathering (Campbell et al., 2009), which may also contribute to modify the MSAC. The determination of quantitative relationships between climate and biomass can be used in predictive models to estimate the influence of climate change on standing biomass and C stock (Cox et al., 2000; Stegen et al., 2011).

Despite the above considerations, relatively little is known about the abiotic environmental factors (including climate) that control the aboveground carbon of forested ecosystems at the stand level (Stegen et al., 2011). We still lack a good understanding of the environmental drivers that determine the forest C stock (Bellassen & Luyssaert, 2014). Selmants et al. (2014) recently reported that live biomass C did not vary predictably as a function of mean annual temperature in tropical montane wet forests on the Island of Hawaii. Stegen et al. (2011) concluded that biomass of forested ecosystems across North, Central and South America was not strongly limited by climate, and that changes in mean climate could not be useful to predict forest biomass changes. In contrast with these two studies, others have documented how forest biomass is influenced by air temperature (Raich, Russell & Kitayama, 2006; Larjavaara & Muller-Landau, 2012), precipitation (Sankaran et al., 2005; Malhi et al., 2006; Saatchi et al., 2007) or the combination of temperature and precipitation regime (Brown & Lugo, 1982; Shuman & Shugart, 2009; Keith et al., 2010; Slik et al., 2010; Liu et al., 2014). Soil properties have also been shown to affect aboveground biomass in the Amazonian forest (Castilho et al., 2006) and on the island of Borneo (Paoli, Curran & Slik, 2008).

Most of the above-mentioned studies were conducted in tropical forests, where absolute temperatures remain within a relatively high range, a cold season is absent, and snow does not accumulate significantly on the ground. Clearly, results obtained in these ecosystems can hardly be extrapolated to temperate and boreal forests. These Nordic ecosystems have received much less attention, despite the fact that they are projected to experience the strongest climate changes over the coming decades (Gauthier et al., 2015). A better understanding of the factors controlling the temperate and boreal forest biomass will improve our capability to predict their response to climate change.

The first objective of this study is to quantify CACS of the managed forest in Quebec, Canada. Our data set includes more than 94,000 forest inventory sample plots distributed throughout a 583,000-km^2^ territory, and spatial data on attributes of the entire managed forest territory interpreted from aerial photographs. The second objective is to build a predictive model of MSAC based on climatic variables. We hypothesized that the latitudinal climatic gradient within the study area is the major factor controlling the MSAC. From these models we aim to project MSAC under present and future climate conditions in order to obtain the first quantitative evaluation of the potential impact of climate change on MSAC over the managed forests of Quebec.

## Material and Methods

### Study area

**Figure 1:**
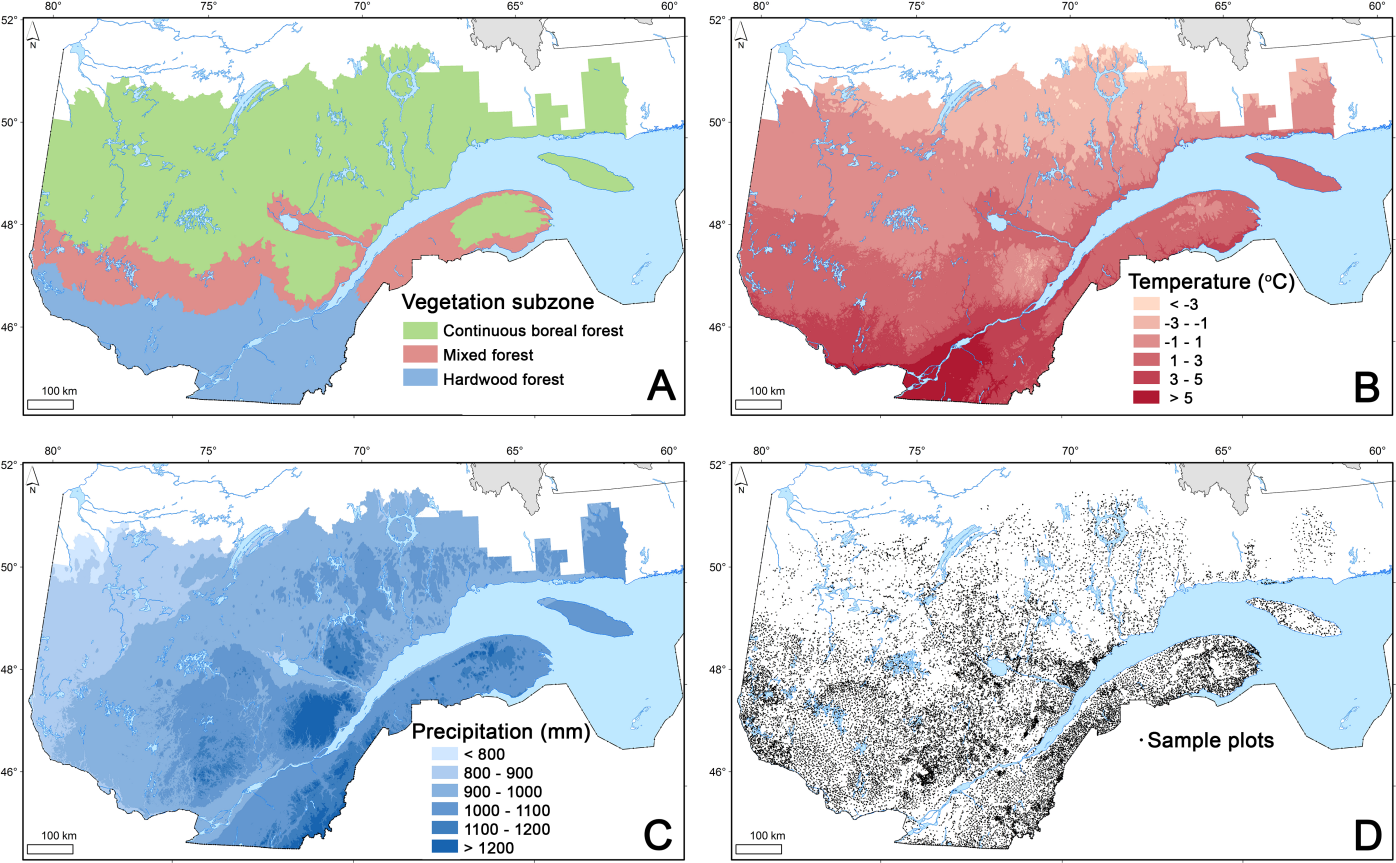
Vegetation subzones of the managed forest in Quebec, Canada (A), mean annual temperature (B), mean annual precipitation (C) and sample plot distribution (D).

The study area corresponds to the forest territory below the current northern limit of the managed forest in Quebec, Canada, which extends from approximately lat. 45° to 52°N and covers approximately 583,078 km^2^, of which 434,667 km^2^ is classified as productive forest. This territory is characterized by three different forest subzones from south to north: the hardwood forest, the mixed forest, and the continuous boreal forest (Fig. 1A, MRN, 2013). The temperate mixed forest (lat. 47°to 48°N) marks the transition between the hardwood forest to the south, which is dominated by sugar maple (*Acer saccharum* Marsh.), and the coniferous forest to the north, which is dominated by balsam fir (*Abies balsamea* [L.] Mill) and black spruce (*Picea mariana* (Mill) B.S.P.). This mixed forest corresponds to the yellow birch (*Betula alleghaniensis* Britt.)—balsam fir bioclimatic domain. The study area is mainly characterized (51.4%) by medium-textured mineral soil deposits thicker than 25 cm and a xeric to mesic soil moisture regime (soil class 2, Table 1). Data from 387 weather stations distributed throughout the studied territory indicated that normal (1971–2000) mean annual temperature ranges from approximately from −2.6 °C to 7.4 °C, and annual precipitation, from 770 mm to 1,600 mm (Figs. 1B and 1C).

**Table 1:** Schematic classification of soil physical environments encoutered in Quebec, numbered from 0 to 9. The proportion (%) of managed forest area corresponding to each class is shown in paretheses.

	Mineral soil				Organic soil
Soil moisture regime	Very shallow (<25 cm) or very stony	Coarse texture	Medium texture	Fine texture	
Xeric to Mesic		1 (8.8)	2 (51.4)	3 (6.7)	
Hygric	0 (10.3)	4 (1.8)	5 (10.5)	6 (4.7)	
Hydric			7 (1.2)		8 – Fen (2.0) 9 – Bog (2.5)

**Notes.**

Xeric Dry, little moisture retention, excessively drained. Water removed very rapidly in relation to supply; soil is moist for brief periods following precipitation Mesic Moist, adequate soil moisture retention year-round. Water removed somewhat slowly in relation to supply; soil may remain moist for a significant, but sometimes short, period of the year. Available soil moisture reflects climatic inputs Hygric Water removed slowly enough to keep soil wet for most of the growing season; permanent seepage and mottling; gleyed (greenish-blue-grey) mottles common in the soil profile Hydric Wet; periodically or often flooded by water. Water removed so slowly that water table is at or above soil surface all year; gleyed mineral or organic soils

The intensity of logging activities in Quebec has changed spatially through time. At the beginning of the 20th century, they were confined to the southern portion of the province (south of lat. 49°N), whereas they currently take place up to lat. 51°N. Besides forest management activities, fire and spruce budworm (*Choristoneura fumiferana* Clemens) outbreaks were the main disturbances regulating forest dynamics over the last century (Girard, Payette & Gagnon, 2008).

### Area-weighted average current carbon stock

Within a conventional forest map, the productive forestland is stratified into polygons representing stands with different forest attributes (composition, density, age, height) and site characteristics (soil deposit, drainage, slope) that are interpreted from aerial photographs (MRNF, 2009a). Forest attributes (number of stems by species and 2 cm diameter class) for each polygon were estimated from the compiled information of the corresponding forest inventory stratum (see next section). Provincial forest managers in Quebec have elaborated a mapping system (*Système d’information forestière par tesselle*; SIFORT) in which a conventional forest map (vector or object-oriented images) is translated into a grid of tiles (mixed vector and raster images) separated by 15″ (∼375 m) (MRNF, 2007). The latest forest map divided the territory in approximately 7.7 million of polygons while the systematic sampling of the whole area results in approximately 4.1 million tiles, of which approximately 3.1 million were classified as productive forests. Land use, site, and forest attributes of each tile correspond to the information of the polygon of the conventional forest map at the center of the tile. This systematic sampling of the conventional forest map results in a relatively high-definition raster map of forest attributes at the provincial scale which has been used to portray the contemporary evolution of the managed forest in southern Quebec over the last three rounds of forest inventory (MRNF, 2009b).

To assess the contemporary area-weighted average and total CACS in live tree biomass for the studied territory, we estimated CACS for each tile characterizing the studied territory based on the third round of forest inventory (1990–2002). We estimated the biomass of standing live trees using the Canadian national species-specific allometric aboveground biomass equations developed by Lambert, Ung & Raulier (2005), then converted the biomass data to C by assigning a C content of 0.5 Mg C per Mg oven dry biomass (IPCC, 2006). We estimated the total C stock in aboveground live biomass by summing the C content of each living stem and reporting the cumulative value on a hectare basis.

### Carbon stock estimates at the plot level

Forest inventory programs conducted by the provincial forest authorities use temporary sample plots (TSPs) to portray existing forest resources comprehensively. The third round of forest inventory (1990–2002) provided data from more than 94,000 TSPs (Fig. 1D). Forest inventory of productive forest over the entire territory followed a stratified sampling design with proportional allocation. Forest stands interpreted from aerial photographs were first stratified based on stand characteristics (composition, density, height, age), edaphic properties (slope, drainage, deposit), and perturbations history (MRNF, 2009a). Circular plots (radius = 11.28 m, area = 400 m^2^) were then proportionally allocated in each strata according to their respective surface area (MRN, 2001). Within each plot, every tree with a diameter at breast height (DBH, measured 1.3 m above the highest root) larger than 90 mm was classified according to its species, and its DBH was measured to the nearest 2-cm class using a tree caliper. The number of saplings (11 mm ≤ DBH ≤ 90 mm) for each 2-cm class was also recorded by species in a subplot (radius = 3.57 m, area = 40 m^2^) (MRN, 2001). We estimated the biomass of standing live trees using the same method employed for C stock estimation at the scale of the forest map (see previous section).

### Influence of climate and soil physical environment on MSAC

We simulated mean annual temperature and precipitation (1971–2000) for each plot with the stochastic weather generator of BioSIM software (Régnière, 1996). Its simulation models provide forecasts based on regional air temperature and precipitation, interpolated from nearby weather stations and adjusted for elevation and location differentials with regional gradients.

Climate appears to be the first factor determining forest biomass and forest distribution at the global scale. However, at the finer scales of landscapes and stands, other factors such as edaphic properties, species interactions and disturbances become important (Pan et al., 2013). Studied plots included widely variable stands in terms of age, structure, composition, and disturbance history. Many of the studied stands had been recently disturbed by forest management activities or natural disturbances and were not fully stocked, since they had not reached maturity. To document the influence of climate and soil physical environment on MSAC, we first stratified sampling plots into a total of 80 strata according to their soil physical environment (10 classes, Table 1), temperature (4 classes; <1 °C, 1–1.9 °C, 2–2.9 °C, ≥3 °C) and precipitation (2 classes; <1,000 mm yr^−1^ and ≥1,000 mm yr^−1^). Secondly, we selected the stands with C stock within the 95% confidence interval of the 90th percentile (Hahn & Meeker, 1991) of the C stock distribution for each of the 80 strata, assuming that they were mature, fully stocked stands in which CACS corresponds to the MSAC. We computed percentiles and related confidence intervals with the CAPABILITY procedure (SAS Institute Inc., 2011). Using this selection, we analyzed multiple regression models to investigate the relationships between mean annual temperature, annual precipitation and MSAC for each of the 10 soil classes. In order to determine the maximum amount of MSAC variance that could be explained as a function of temperature, precipitation, their squared value, and their interaction, we tested all possible subsets regression models with the REG procedure (SAS Institute Inc., 2011). We selected candidate models on the basis of two widely used criteria: Mallow’s Cp-statistic (Mallows, 1973) and Akaike’s information criteria (Akaike, 1973). Only subset models that had Mallow’s Cp values close to the number of parameters were considered. The final model selection was based on the lowest AIC. We tested for multicollinearity among the selected models’ climate variables using condition indexes and the variance inflection factor, to verify that dependencies among temperature and precipitation did not affect the regression estimates (Belsley, Kuh & Welsch, 1980). We also checked residuals graphically to assess the tenability of the regression assumptions.

### The impact of climate change on MSAC

To assess the area-weighted average MSAC response to anticipated climate change, we compared the MSAC predicted from climate data under present and future climate scenarios using previously calibrated models at the plot level. For the present climate scenario, we used the BioSIM software (Régnière, 1996) to compute mean annual temperature and precipitation (1971–2000) for each tile. For the future climate scenario, we obtained climate projections for each tile using the delta change method (Fowler, Blenkinsop & Tebaldi, 2007) calculated from the output of global climate models (GCM), then applied them to the observed values for each tile. Future values were constructed using a large ensemble of GCMs made available by the World Climate Research Programme’s (WCRP’s) Coupled Model Intercomparison Project phase 3 (CMIP3) multi-model dataset (Meehl et al., 2007). Simulated climate data was available for present-day (20th century) atmospheric conditions and for projected future climate in response to three projected future greenhouse gas emission scenarios (SRES family A2, A1B and B1 scenarios; Nakicenovic et al., 2000) which have been endorsed by the IPCC and form the basis of their 4th assessment report (AR4) published in 2007. More recently, the publication of the IPCC 5th assessment in 2013 has caused a general shift away from the use of CMIP3 data in impact studies. This shift was particularly marked by a replacement of SRES greenhouse gas emissions scenarios by Representative Concentration Pathways (Moss et al., 2010) or RCPs. AR4 results remain valid however and comparisons between RCP and the SRES family scenarios can been made. Approximate equivalents (in terms of average global temperature) can be seen from the results of Knutti & Sedláček (2012) where SRES B1 is roughly equivalent to RCP 4.5 and SRES A1B is most similar to RCP 6.0, while SRES A2 is seen to be somewhere between RCP 6.0 and 8.5, showing stronger warming than RCP 6.0 but not as strong as RCP 8.5. Gleckler, Taylor & Doutriaux (2008) demonstrated that using the median or average of a large ensemble of climate simulations was more robust than using individual projections to reproduce the observed climate. This approach also has the advantage of providing a more robust indication of uncertainty of the projected future conditions. In all, we selected 68 climate simulations for two future horizons: 2041–2070 and 2071–2100.

As previously mentioned for the actual MSAC, our assessment of the future MSAC does not take into account the potential changes in natural disturbance regimes and forest management practices as well as the potential effects of increasing CO_2_ concentration and nitrogen deposition.

### Uncertainty analysis

Raupach et al. (2005) and Keith et al. (2010) identified many potential sources of errors that may cause uncertainties in C accounting across heterogeneous landscapes. These include the extent to which the field sampling covers the diversity of forest ecosystems across the landscape, error associated to the spatial extrapolation of site data, accuracy of tree allometric equations for estimating tree biomass, and variability in the C to biomass ratio of trees. We apportioned the main sources of uncertainty related to area-weighted average CACS and MSAC estimates with Monte Carlo uncertainty analysis (Yanai et al., 2010). First, we documented uncertainty due to tree biomass equations based on national forest inventory data by randomly sampling allometric model parameters and residual variance estimates values from their specified distribution (100 iterations, Lambert, Ung & Raulier, 2005) to generate a distribution of tree biomass estimates. We limited our analysis to 100 iterations because of the very large data set involved (171.8 million records). From these results, we calculated the mean total biomass and its standard deviation. Then, we generated a distribution of CACS estimates by combining total biomass and wood C content variability of North American hardwood and softwood species (10,000 iterations, Lamlom & Savidge, 2003).

Secondly, we documented the uncertainty related to the MSAC modeled from temperature and precipitation data along with uncertainty associated with future climate scenarios (100 iterations, Logan et al., 2011) to assess these sources of uncertainty related to area-weighted average estimates of MSAC under present and future climate. Uncertainty was reported as the coefficient of variation (standard deviation divided by the mean) of the distributions of Monte Carlo iterations. Our uncertainty analysis focused on the main sources of uncertainty related to CACS and MSAC computation, but did not take into account uncertainty in forest inventory measurements, their up-scaling to the entire territory, or the uncertainty associated to the interpretation of stand and site characteristics from aerial photography.

**Figure 2:**
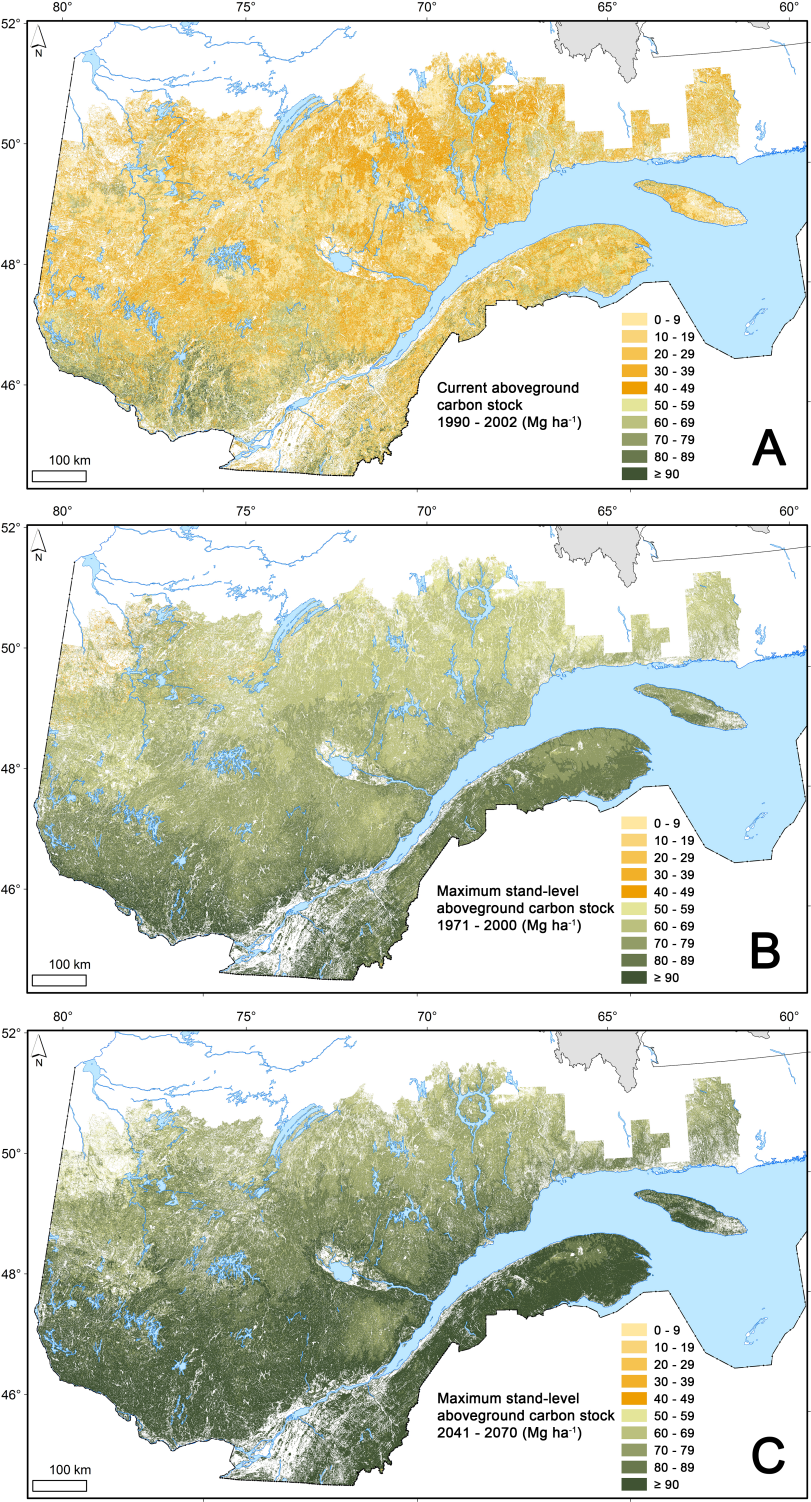
Current and maximum stand-level aboveground carbon stock. Estimates of current carbon stock (1990–2002) in aboveground live biomass (A) and of maximum stand-level aboveground carbon stock under present (1971–2000), (B) and future (2041–2070), (C) climate scenarios, mapped at a ∼375 m resolution from a combination of forest inventory data and aerial photographs.

**Table 2:** Current and maximum stand-level aboveground carbon stock. Estimates of area-weighted average current aboveground carbon stock and maximum stand-level aboveground carbon stock (Mg ha^−1^ ± SD^a^) (total (Tg) in parentheses) in the three subzones of the managed forest of Quebec, Canada, under present climate and future climate scenarios.

	Current carbon stock in aboveground live biomass	Maximum stand-level aboveground carbon stock		
		1971–2000	2041–2070	2071–2100
Hardwood forest	52.9 ± 26.1 (392.2)	88.3 ± 9.8	108.7 ± 12.8	119.7 ± 14.6
Mixed forest	41.2 ± 22.3 (327.6)	78.0 ± 7.1	95.1 ± 9.2	104.4 ± 10.3
Continuous boreal forest	32.5 ± 21.6 (913.2)	68.8 ± 7.2	75.8 ± 10.1	85.8 ± 11.6
Total managed forest	37.6 ± 23.7 (1,633.0)	72.5 ± 11.2	86.7 ± 15.6	95.0 ± 17.8

**Notes.**

a SD: Standard deviation from tiles.

**Figure 3:**
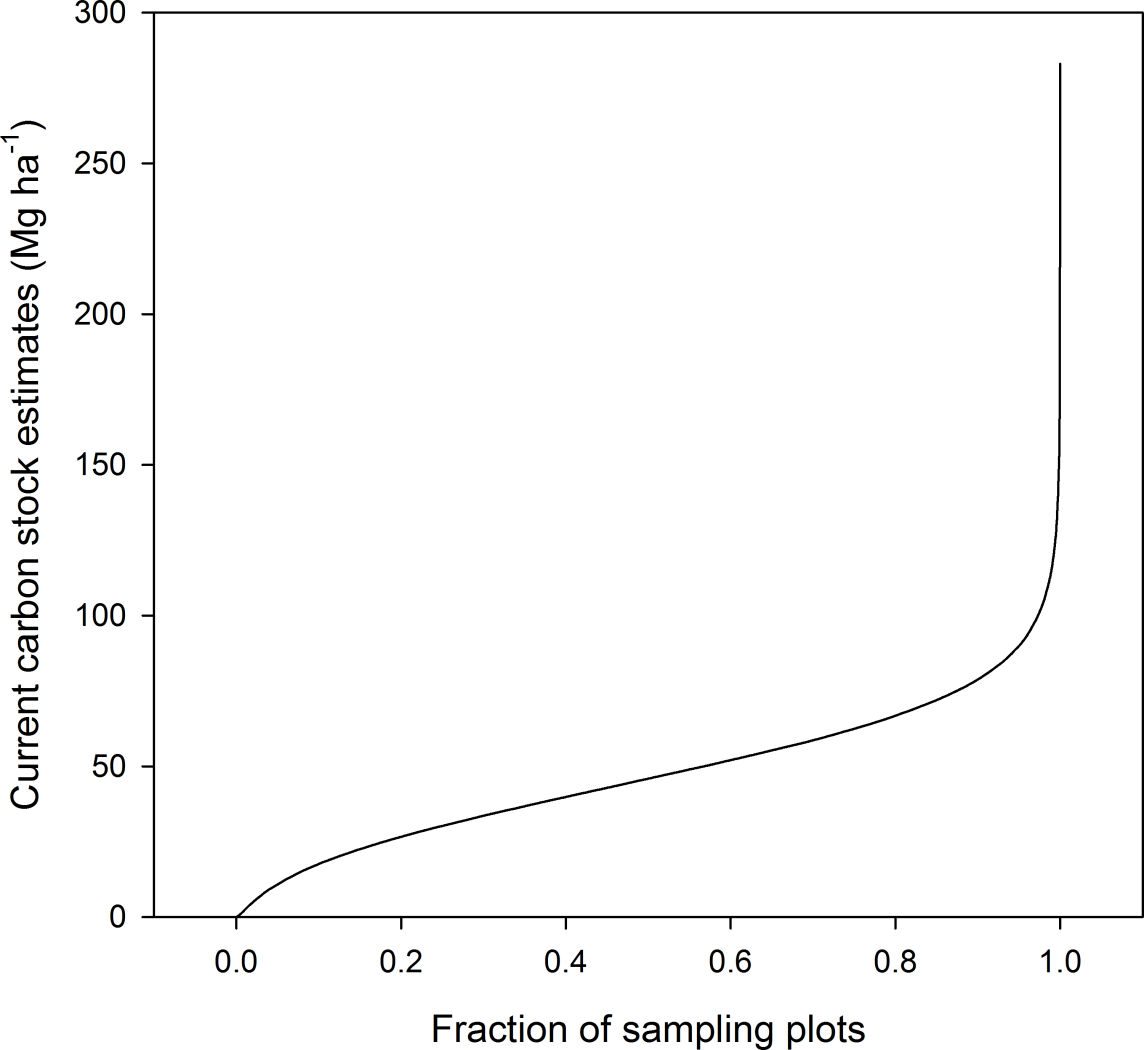
Quantile plot of the current carbon stock estimates in the aboveground live biomass of forest sample plots.

## Results

### Current aboveground carbon stock estimates

CACS in live tree biomass for the whole 43.5-M ha study area (Fig. 2A) was estimated to be 1,633 Tg of which, because of its size, the boreal forest alone represents 56% (Table 2). This represents an area-weighted average CACS of 37.6 Mg ha^−1^, including only land classified as productive forest. On average, forest stands in the hardwood subzone stocked approximately 41% more C per ha than those in the boreal forest ecozone. The area-weighted average CACS decreases from the hardwood forest in the south to the mixed and continuous boreal forests in the north (Table 2).

In sampling plots, CACS averaged 47.8 Mg ha^−1^ and ranged from 0.03 to 100 Mg ha^−1^ in 97.3% of the 94,268 forested sampling plots, although some reached exceptional values of up to 283.1 Mg ha^−1^ (Fig. 3). The 18 most stocked stands (>200 Mg ha^−1^) included old Norway spruce (*Picea abies* [L.] H. Karst.) plantations (one of the first non-native species introduced to North America) (39%), uneven-aged mature sugar maple stands (39%), and mature quaking aspen (*Populus tremuloides* Michaux) stands (22%). Although CACS was highly variable spatially, it was larger in warmer sites in the south of the province than in more northerly sites (Table 3). On average, CACS increased from 41.3 Mg ha^−1^, for sites within the colder temperature class (<1 °C), to 58.0 Mg ha^−1^ for sites within the warmer class (≥3 °C). On average, CACS was also slightly larger (49.6 Mg ha^−1^) on wetter (≥1,000 mm) than on drier sites (<1,000mm, 45.3 Mg ha^−1^). On average, the highest CACS values were observed on xeric to mesic sites with fine to medium-textured soils (soil class 2 and 3), while the lowest values were observed on hydric mineral soils and organic soils (soil class 7, 8, and 9).

**Table 3:** Average current carbon stock in sample plots. Current carbon stocks in aboveground live biomass, expressed in Mg ha^−1^ ± SD^a^ (number of plots in parentheses), according to each climate-soil class stratum.

Climate classes		Soil class^b^										
Air temp. (°C)	Precip. (mm)	0	1	2	3	4	5	6	7	8	9	Average
<1	<1,000	40.8 ± 17.9 (1,034)	36.8 ± 18.2 (1,717)	42.3 ± 19.8 (9,114)	47.8 ± 23.7 (442)	38.5 ± 17.5 (215)	40.4 ± 18.5 (2,203)	39.8 ± 22.7 (1,033)	34.8 ± 16.0 (482)	33.7 ± 17.2 (456)	30.1 ± 15.8 (920)	**40.3 ± 19.6(17,616)**
1–1.9		39.0 ± 18.9 (1,302)	38.9 ± 20.3 (1,153)	44.5 ± 21.6 (4,687)	46.2 ± 23.8 (484)	34.4 ± 19.6 (164)	36.6 ± 18.2 (934)	38.9 ± 21.4 (535)	31.3 ± 15.6 (226)	28.8 ± 16.3 (273)	29.2 ± 15.2 (409)	**40.7 ± 21.0(10,167)**
2–2.9		46.0 ± 22.3 (1,081)	42.0 ± 22.7 (743)	57.7 ± 26.4 (4,254)	47.0 ± 25.2 (152)	40.7 ± 20.0 (121)	47.4 ± 22.4 (638)	38.9 ± 20.8 (136)	33.9 ± 16.8 (75)	37.0 ± 19.9 (230)	33.2 ± 16.9 (249)	**51.2 ± 25.7(7,679)**
≥3		60.7 ± 25.8 (717)	55.2 ± 26.4 (726)	66.5 ± 27.0 (2,912)	64.5 ± 29.7 (153)	51.8 ± 24.6 (212)	54.1 ± 24.4 (442)	50.0 ± 27.5 (157)	40.5 ± 27.5 (38)	50.0 ± 23.2 (306)	41.6 ± 21.8 (75)	**60.1 ± 27.2(5,738)**
<1	≥1, 000	38.5 ± 18.4 (777)	37.2 ± 19.4 (423)	43.8 ± 20.2 (7,538)	45.0 ± 21.7 (784)	37.8 ± 16.7 (116)	43.0 ± 18.5 (1,632)	46.8 ± 20.7 (97)	35.5 ± 15.8 (118)	33.9 ± 18.0 (154)	32.9 ± 16.9 (333)	**42.6 ± 20.0(11,942)**
1–1.9		47.3 ± 23.0 (979)	41.0 ± 19.5 (508)	48.5 ± 23.1 (7,869)	48.5 ± 23.4 (2,080)	41.5 ± 19.9 (100)	42.1 ± 21.7 (1,282)	45.2 ± 22.3 (475)	36.9 ± 18.0 (94)	37.8 ± 23.7 (415)	36.1 ± 19.5 (280)	**46.8 ± 23.0(14,082)**
2–2.9		52.6 ± 22.6 (916)	43.4 ± 23.2 (322)	55.9 ± 26.5 (7,141)	52.2 ± 26.2 (2,255)	38.2 ± 20.5 (53)	45.5 ± 24.0 (1,301)	42.6 ± 23.7 (762)	33.3 ± 19.2 (86)	41.9 ± 22.0 (705)	39.2 ± 19.5 (294)	**51.8 ± 25.9(13,835)**
≥3		61.4 ± 25.9 (784)	54.7 ± 30.0 (486)	63.2 ± 28.7 (5,084)	58.8 ± 27.7 (1,484)	48.4 ± 27.8 (424)	52.2 ± 25.8 (2,003)	52.2 ± 25.7 (1,320)	40.6 ± 23.5 (246)	47.3 ± 23.4 (1,082)	37.7 ± 20.0 (286)	**56.8 ± 27.9(13,209)**
**Average**		**47.3 ± 23.2(7,590)**	**42.2 ± 22.8(6,078)**	**50.7 ± 25.0(48,599)**	**51.3 ± 25.6(7,814)**	**43.4 ± 23.5(1,405)**	**44.6 ± 22.2(10,435)**	**44.8 ± 24.5(4,515)**	**35.5 ± 18.5(1,365)**	**41.0 ± 22.4(3,621)**	**33.2 ± 17.7(2,846)**	**47.8 ± 24.5(94,268)**

**Notes.**

a SD: Standard deviation.

b As defined in Table 1.

### MSAC and their relationships with climate

Table 4 presents the average CACS of the selected fully stocked stands (within the 95% confidence interval of the 90th percentile of the C stock distribution, and assumed to correspond to the MSAC) according to each stratum. The selected stands represent 2.9% of all plots. They vary widely in terms of composition (28% hardwood, 27% mixed, 45% coniferous), are mostly (90%) higher than 12 m, and generally (95%) have more than 40% forest cover. Depending on the stratum, average CACS of the fully stocked stands were 51.4–89.6% larger and generally less variable than CACS estimated from all plots (Tables 3 and 4). As previously mentioned, we assumed that these stands had reached their MSAC with respect to the present climate and their soil physical environment.

**Table 4:** Average carbon stocks of the fully-stocked stands. Average carbon stocks in aboveground live biomass, expressed in Mg ha^−1^ ± SD^a^ (number of plots in parentheses), of the fully-stocked stands (carbon stocks within the 95% confidence interval of the 90th percentile, corresponding to the maximum stand-level aboveground carbon stock) for each climate-soil class stratum.

Climate classes		Soil classes^b^										
Air temp. (°C)	Precip. (mm)	0	1	2	3	4	5	6	7	8	9	Average
<1	<1,000	63.8 ± 1.2 (39)	62.2 ± 1.4 (50)	68.5 ± 0.4 (114)	76.8 ± 2.0 (26)	61.9 ± 2.9 (19)	64.7 ± 0.8 (57)	71.4 ± 1.4 (39)	55.6 ± 1.8 (27)	57.2 ± 1.3 (27)	51.3 ± 1.4 (37)	**64.4 ± 6.6(435)**
1–1.9		63.1 ± 1.5 (44)	64.0 ± 1.5 (41)	72.1 ± 0.9 (82)	78.1 ± 2.0 (27)	60.7 ± 6.1 (17)	61.5 ± 1.4 (37)	65.4 ± 2.1 (29)	51.0 ± 2.4 (19)	48.4 ± 2.5 (21)	49.0 ± 1.9 (25)	**63.8 ± 8.8(342)**
2–2.9		74.2 ± 2.5 (40)	72.2 ± 2.4 (34)	93.1 ± 1.1 (78)	78.9 ± 4.9 (16)	68.3 ± 9.1 (14)	76.4 ± 2.0 (31)	65.6 ± 3.9 (15)	57.7 ± 7.5 (12)	63.8 ± 4.2 (19)	55.1 ± 2.4 (20)	**76.2 ± 12.7(279)**
≥3		94.0 ± 3.1 (33)	86.2 ± 1.9 (33)	100.7 ± 1.0 (65)	100.6 ± 8.5 (16)	85.4 ± 3.7 (19)	85.8 ± 2.3 (26)	85.2 ± 5.2 (16)	76.8 ± 23.7 (9)	79.2 ± 2.6 (22)	72.5 ± 9.1 (12)	**90.1 ± 10.4(251)**
<1	≥1, 000	62.7 ± 1.4 (34)	62.3 ± 1.9 (26)	70.3 ± 0.4 (104)	73.3 ± 1.3 (34)	60.6 ± 3.6 (14)	66.5 ± 1.1 (49)	71.7 ± 4.3 (13)	55.4 ± 1.8 (14)	56.9 ± 3.7 (16)	54.6 ± 2.4 (23)	**65.8 ± 6.1(327)**
1–1.9		76.3 ± 1.3 (38)	68.1 ± 2.2 (28)	77.1 ± 0.6 (106)	77.8 ± 1.2 (55)	69.8 ± 4.7 (13)	70.7± 1.5 (44)	75.4 ± 2.7 (27)	61.7 ± 3.5 (13)	68.1 ± 1.7 (25)	61.2 ± 3.2 (21)	**73.3 ± 5.5(370)**
2–2.9		81.8 ± 1.8 (37)	74.6 ± 3.3 (23)	89.2 ± 0.6 (101)	86.0 ± 1.4 (57)	70.0 ± 12.2 (10)	77.2 ± 1.6 (44)	73.8 ± 2.6 (34)	59.3 ± 5.4 (12)	71.1 ± 2.6 (33)	63.6 ± 3.2 (22)	**79.7 ± 9.1(373)**
≥3		95.2 ± 2.1 (34)	93.2 ± 4.2 (27)	99.1 ± 1.0 (85)	92.8 ± 2.0 (47)	82.3 ± 3.9 (26)	84.4 ± 1.2 (54)	82.3 ± 1.5 (44)	71.2 ± 3.7 (20)	75.5 ± 1.2 (40)	63.8 ± 1.7 (21)	**87.1 ± 10.4(398)**
**Average**		**75.7 ± 12.3(299)**	**71.7 ± 10.9(262)**	**82.2 ± 12.2(735)**	**82.8 ± 8.2(278)**	**71.1 ± 11.2(132)**	**72.8 ± 8.4(342)**	**74.3 ± 6.9(217)**	**60.1 ± 10.4(126)**	**66.5 ± 9.8(203)**	**57.3 ± 7.4(181)**	**74.5 ± 12.8(2,775)**

**Notes.**

a SD: Standard deviation.

b As defined in Table 1.

**Figure 4:**
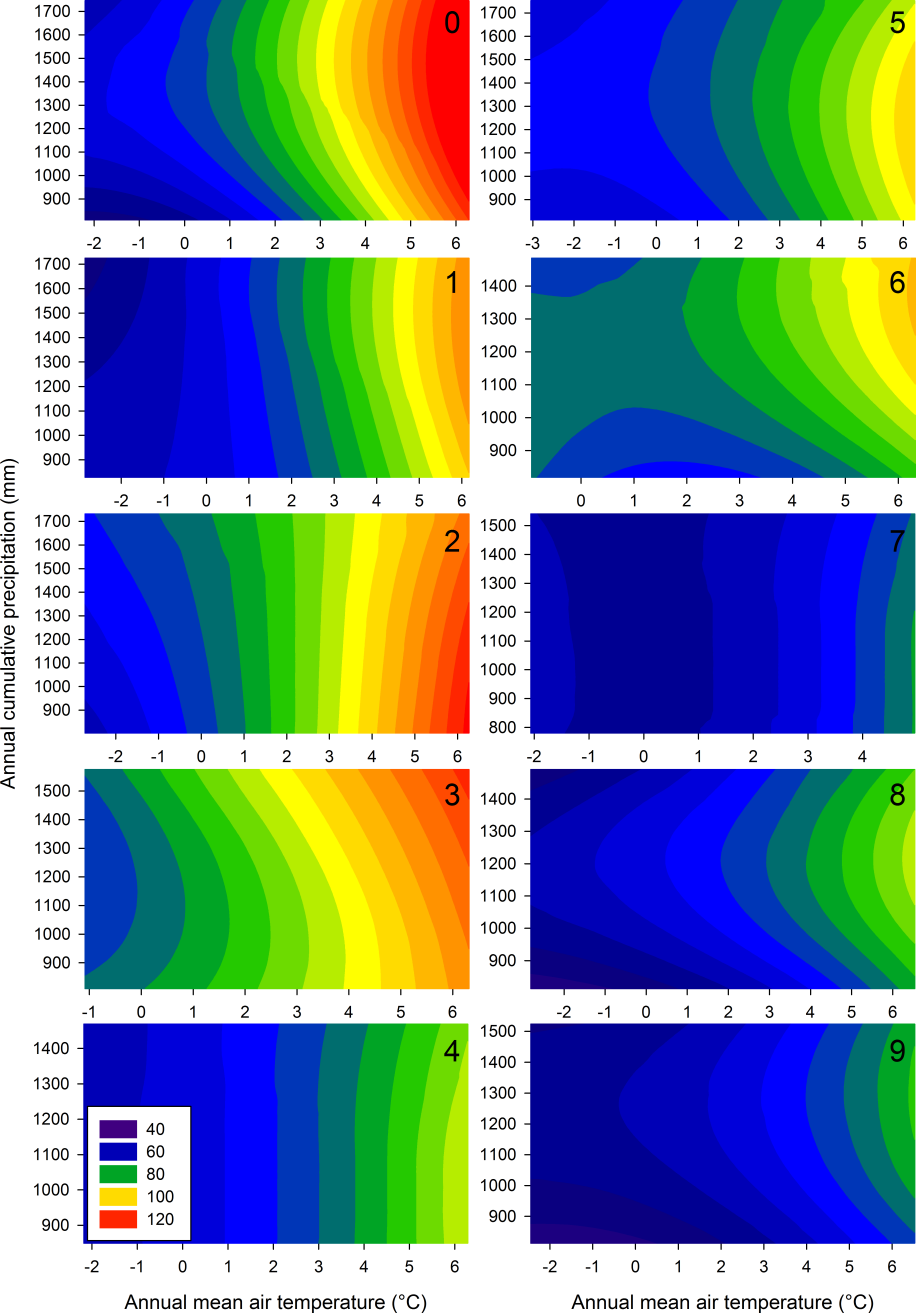
Effect of climate on the maximum stand-level aboveground carbon stock. Contour plots summarizing the effect of mean annual precipitation and temperature under the present climate on the maximum stand-level aboveground carbon stock (Mg ha^−1^) of forest ecosystems in Quebec, Canada. Numbers in the upper right corners refer to the soil classes defined in Table 1.

**Figure 5:**
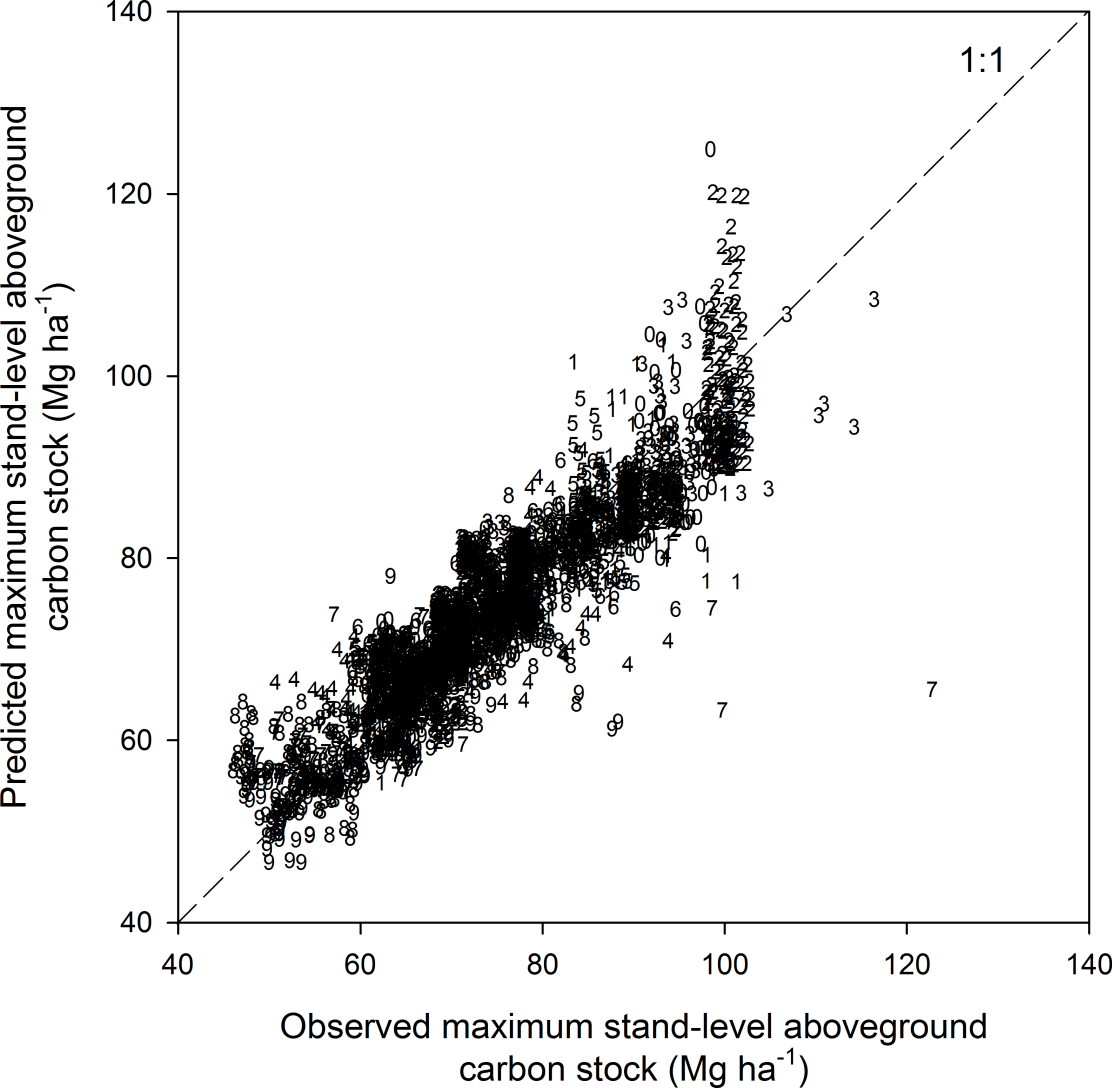
Observed vs. predicted maximum stand-level aboveground carbon stock of forest ecosystems (*N* = 2,775). The numbers refer to the soil classes defined in Table 1. See Table 4 for model statistics.

The MSAC was significantly related to both mean annual temperature and precipitation, or to the interaction between these variables (Table 5), for all soil classes except hydric mineral soils (soil class 7) and hygric coarse-textured soils (soil class 4), which were not related significantly to precipitation. Temperature and precipitation generally explained over 60% of the variance in MSAC, except for hydric mineral soils (40%, soil class 7) and bogs (48%, soil class 9). Overall, most of the variance explanation is due to the temperature gradient (Fig. 4). The explained variance was also higher (74–81%) for the soil classes 0–3 and 5, which cover the major proportion of the studied area as compared to the more marginal sites (40–64% of variance explanation for soil classes 4, 6, 7, 8, and 9) (Table 5). Overall, observed and predicted MSAC values for all the observations (*n* = 2, 775) were tightly correlated and well distributed along the 1:1 line (correlation coefficient = 0.91, Fig. 5).

**Table 5:** Regression analysis. Results of the regression analysis relating the maximum stand-level aboveground carbon stock of various soil classes to mean annual temperature (*T*, °C) and precipitation (*P*, mm).

Soil classes^a^	Coefficient (*P*-values in parentheses) for the variables included in the models						*R*^2^	CV^b^ %
	Intercept	*T* linear	*T* quadratic	*P* linear	*P* quadradic	*T* × *P*		
0	−16.35 (0.222)	4.272 (<0.001)	0.889 (<0.001)	0.123 (<0.001)	−4.4E–5 (<0.001)		0.81	7.2
1	61.96 (<0.001)		0.581 (<0.001)			0.003 (<0.001)	0.80	6.9
2	65.60 (<0.001)	7.293 (<0.001)	0.463 (<0.001)	0.005 (0.004)		−0.002 (0.019)	0.78	6.9
3	113.5 (<0.001)		0.321 (<0.001)	−0.072 (0.001)	3.2E–5 (0.002)	0.004 (<0.001)	0.74	5.1
4	61.58 (<0.001)	2.168 (0.002)	0.421 (0.001)				0.64	9.5
5	32.61 (0.001)	2.126 (<0.001)	0.475 (<0.001)	0.052 (0.002)	−1.9E–5 (0.006)		0.75	5.9
6	23.23 (0.178)	−7.955 (<0.001)	0.759 (<0.001)	0.086 (0.009)	−3.7E–5 (0.014)	0.006 (<0.001)	0.63	5.7
7	54.53 (<0.001)		0.895 (<0.001)				0.40	13.5
8	−57.47 (0.035)	2.442 (<0.001)	0.254 (0.013)	0.199 (<0.001)	−8.2E–5 (0.001)		0.61	6.2
9	−17.6 (0.399)	1.497 (0.002)	0.333 (0.004)	0.117 (0.002)	−4.7E–5 (0.007)		0.48	5.4

**Notes.**

a As defined in Table 1.

b CV = Coefficient of variation calculated as the ratio of the root mean squared error (RMSE) to the mean of the dependent variable.

### MSAC response to climate change

Based on the previously defined MSAC models presented in Table 5, area-weighted average MSAC of individual stands over the whole territory (under the present climate: 1971–2000) was estimated to 72.5 Mg ha^−1^ (Table 2, Fig. 2B). On average, future climate projections for the study area propose a median increase of air temperature of 2.4 °C in 2041–2070 and of 3.4 °C in 2071–2100 (Table 6). Concurrently, median annual precipitation is expected to increase by 7.5% in 2041–2070 and by 10.5% in 2071–2100. Based on the previously-defined models that relate MSAC and climate, under future climate scenarios the MSAC is expected to increase, on average by 20% in 2041–2070 and by 31% in 2071–2100 (Table 2, Fig. 2C). Regardless of the study period, average MSAC values on a per ha basis decreased from south to north and from warmer to colder climates, with the greatest values found in the hardwood forest subzone, followed by the mixed forest and continuous boreal forest (Table 2).

**Table 6:** Average mean annual temperature and annual precipitation. Estimates of average mean annual temperature (°C ±SD^a^) and annual precipitation (mm ±SD^a^) in the three subzones of the managed forest of Quebec, Canada, under present and future climate scenarios.

	Mean annual temperature			Annual precipitation		
	1971–2000	2041–2070	2071–2100	1971–2000	2041–2070	2071–2100
Hardwood forest	3.5 ± 1.1	5.8 ± 1.0	6.9 ± 1.0	1,031 ± 104	1,099 ± 109	1,124 ± 107
Mixed forest	1.9 ± 0.6	4.3 ± 0.6	5.4 ± 0.5	1,012 ± 75	1,083 ± 77	1,111 ± 76
Continuous boreal forest	−0.3 ± 1.2	2.1 ± 1.2	3.2 ± 1.2	981± 105	1,058 ± 109	1,090 ± 109
Total managed forest	0.7 ± 1.8	3.1 ± 1.8	4.2 ± 1.8	995 ± 102	1,069 ± 105	1,099 ± 105

**Notes.**

a SD: Standard deviation from tiles.

### Uncertainty analysis

Uncertainty related to total and area-weighted average estimates of CACS live tree biomass due to allometric equations was 0.4%, while uncertainty related to average wood C-biomass fraction was estimated to 0.9%. When both sources of uncertainty were combined, uncertainty related to total (1,633 Tg C, Table 2) and area-weighted averages CACS estimates (37.6 Mg ha^−1^, Table 2) was 1.0%. These results are coherent with reported uncertainty of 1% for total wood volume estimates over the same territory (Commission d’étude sur la Gestion de la Forêt publique Québécoise, 2004; Bureau du Forestier en Chef, 2013). The uncertainty related to the MSAC modeled from current temperature and precipitation data was 0.4% while the uncertainty associated with future MSAC estimates was 5.4% and 9.9% for the 2041–2070 and the 2071–2100 periods, respectively. Clearly, the variability among the climate scenarios was the main source of uncertainty in future MSAC estimates.

## Discussion

### Current aboveground carbon stock estimates

In the last forest resources assessment report of the Food and Agriculture Organization of the United Nations (FAO, 2010) the average CACS in live biomass of the Canadian managed forest was estimated to 45 Mg ha^−1^, while the Intergovernmental Panel on Climate Change reported an average aboveground C stock of 48 Mg ha^−1^ for the American temperate and coniferous forest (excluding the forest tundra and very young forests (<20 years old), and assuming half of the biomass is C; IPCC, 2003). According to Fang et al. (2006) who conducted a literature review, inventory-based forest C stocks in middle and high northern latitudes ranged from 36 to 56 Mg ha^−1^, and averaged 43.6 Mg ha^−1^. These estimates are very close to the average C stock of 47.8 Mg ha^−1^ we estimated from the sampling plot network. Our area-weighted average CACS estimate (37.6 Mg ha^−1^), which accounted for spatial heterogeneity in forests and site attributes, was however 21% lower than the average C stock of 47.8 Mg ha^−1^ obtained when not considering spatial heterogeneity and simply averaging the values of the 94,268 sampling plots (these plots followed a stratified, not a random, sampling). Such an evaluation (i.e., one that takes into account the biomass stock for each tile of a static grid covering the entire territory) can be used as a reference to estimate changes in aboveground tree C storage in the future, and also to document the role of forest and management practices on regional C stock and budget.

### MSAC and their relationships with climate

Sampling plots represent a wide range of variability in terms of stand age, structure, composition, productivity, edaphic qualities, and disturbance history. Because of the large number of observations, we were able to categorize the stands within 80 climate–soil physical environment strata. We assumed that selecting only the most stocked stands, within each of these 80 strata, provided a good approximation of the MSAC as constrained by soil physical properties and climatic environment. Analyzing so many strata has the advantage of constraining the potential spatial variability in MSAC that could be associated with site edaphic qualities, productivity, vegetation types and disturbance histories. Nevertheless, some bias can exist for certain specific strata. For instance, on some marginal sites, the MSAC can be limited by non-climatic factors (e.g., edaphic qualities such as soil chemical properties), and thus be overestimated. Conversely, the MSAC can be underestimated for exceptional sites with very large MSAC that might stock more C than the confidence interval formed by the 90th percentile values of the C distribution, for a given climate–soil physical environment stratum. Despite these considerations, we are confident that our selection of the most stocked stands within a specific climatic zone for a given soil class represents a robust approach for estimating the MSAC of forest ecosystems.

The area-weighted average MSAC under the present climate was evaluated at 72.5 Mg ha^−1^, being twice higher than the CACS (37.6 Mg ha^−1^). Indeed, this is clearly a potential difference given that the MSAC definition implies a forest landscape composed of fully stocked mature stands with no natural disturbance regime and human intervention. Each year over the 2007–2012 period, large-scale fires and insect outbreaks respectively affected 0.3% (1,242 km^2^ yr^−1^) and 0.4% (1,831.2 km^2^ yr^−1^ severely defoliated) of Quebec’s managed forestland (Boulay, 2013). At the landscape scale, such a disturbance regime maintains a portion of the forest area in younger age classes. A modelling exercise based on available data of past stand-replacing disturbance histories (fire, spruce budworm outbreaks, windthrow) revealed that under the preindustrial natural disturbance regime, old-growth forests would compose, on average, 65% of the forest area in Quebec (49–86%, depending on the ecoregion considered) (Boucher et al., 2011).

### The relationship between climate and MSAC

In good agreement with previous studies (see ‘Introduction’ section), we also found strong relationships between climate and MSAC for the various soil classes studied (Table 5). Temperature was responsible for most of the variation in MSAC, presumably because precipitation was relatively abundant within most of the studied territory. Overall, our data show the crucial importance of climate for controlling the MSAC of temperate and boreal forests, for which few results were available to date. Our results also suggest that along with climate, soil properties such as moisture regime and texture can influence the MSAC of forest ecosystems and weaken the MSAC–climate relationship. Indeed, MSAC was lower on marginal, poorly drained sites (hydric moisture regime, classes 7, 8 and 9) than on drier soil types. At the other end of the spectrum, fine to medium-textured mineral soil with xeric to mesic moisture regimes (soil classes 2 and 3) exhibited the greatest MSAC.

### Projection of MSAC response to climate change

The projections show that forest MSAC has the potential to increase considerably in response to climate change. Area-weighted average MSAC over the studied territory is projected to increase from 72.5 Mg ha^−1^ in 1971–2000 to 86.7 Mg ha^−1^ in 2041–2070 and to 95.0 Mg ha^−1^ in 2071–2100 (Table 2). Increased temperature is clearly the main driver of the projected increase in forest MSAC under future climate. In relative terms, the projected MSAC increase in 2071–2100 is greater for stands in the hardwood and mixed forests subzones (34–36%) than for stands in the boreal subzone (25%). Although they may appear surprisingly high, MSAC projections for stands in the temperate and boreal subzone over the 2071–2100 period compare well with contemporary MSAC estimates of mature forests located in areas characterized by climatic conditions similar to those expected in 2071–2100 for the studied territory (Hoover, Leak & Keel, 2012; Keith, Mackey & Lindenmayer, 2009; Liu et al., 2014). By 2071–2100, mean annual temperature is expected to reach 6.9 °C in the hardwood subzone with a mean annual precipitation value of 1,124 mm (Table 6). The projected MSAC for the hardwood subzone in 2071–2100 (119.7 Mg ha^−1^) compares very well with the average aboveground C stock of 120.5 Mg ha^−1^ found in live tree biomass of 12 old-growth forest stands in New England, USA (Hoover, Leak & Keel, 2012). Despite regional discrepancies in terms of nitrogen deposition, solar radiation and other physiological constraints, the studied sites in New England were characterized by climatic conditions similar to those expected in 2071–2100 for the hardwood subzone in Québec with mean annual temperature and annual precipitation ranging respectively from 6.1 to 7.6 °C and from 993 to 1,270 mm (Hoover, Leak & Keel, 2012).

The projected increases in MSAC, though quantitatively important, is a potential value that will not be reached. For instance, a part of the projected MSAC increase may require changes in forest composition and soil properties that are unlikely to happen within the next few decades (Lafleur et al., 2010). Also, the projected MSAC values do not take into account potential C losses that could be due to changes in disturbance regimes that may lead to an increase in burnt or defoliated areas, or to increase moisture stress associated to warming (Gauthier et al., 2015). Nevertheless, the large increase in MSAC values projected under future climate point to an important potential for Quebec forests to store more C in biomass in the future. Along with changes in climate and disturbance regime, forest management may have an important impact on future carbon stock at the landscape scale. This should encourage forest management practices that may optimize at the same time the carbon stocking of future forests, the production of long lived forest products, and forest resilience to climate change and natural perturbations.

## Conclusions

We estimated CACS in live biomass of the Quebec’s managed forest from a data set of more than 94,000 forest plots (with measurements made in the 1990–2002 period) and from a large set of spatial data on forest attributes interpreted from aerial photographs. We were able to build the first map of forest CACS with a fine spatial resolution (∼375 m). We estimated the CACS in live biomass of Quebec’s forests at 1,633 Tg, on a total area of 43.5 M ha. This corresponds to an area-weighted landscape average of 37.6 Mg ha^−1^. The area-weighted average MSAC is estimated to 72.5 Mg ha^−1^. Mean annual temperature and, to a lesser extent, annual precipitation, explained a large part of the spatial variation in MSAC throughout the study area. We also used models relating MSAC and mean annual temperature and annual precipitation to estimate forest MSAC under future climate conditions. In relative terms, the projected MSAC increase in 2071–2100 is greater for the hardwood and mixed forests stand (34–36%) than for boreal stands (25%). The important MSAC increase in response to climate change suggests a greater potential to store C in forest biomass in the coming decades, although this potential may not be realized due to increased natural disturbance rates and slow changes in forest composition and soil properties. Despite many potential sources of error, which can cause uncertainty in C accounting across heterogeneous landscapes, large-scale estimates are important to document the influence of forest management and disturbance regimes on C stock in aboveground living tree biomass in the future, and to quantify possible responses to climate change.

## Additional Information and Declarations

### Data Availability

The following information was supplied regarding data availability: Figshare: https://figshare.com/s/8f901efdaec99f220f09.
